# A Case Report of Cryoneurolysis for Dorsal Foot Pain and Toe Clawing in a Patient With Multiple Sclerosis

**DOI:** 10.1016/j.arrct.2023.100286

**Published:** 2023-07-28

**Authors:** Fraser MacRae, Abby Speirs, Andrei Bursuc, Mahdis Hashemi, Paul Winston

**Affiliations:** a Western University, Faculty of Health Sciences, London, Canada; b Vancouver Island Health Authority, Victoria, Canada; c Centre Hospitalier de l'Université de Montréal, Montréal, Canada; d University of British Columbia, Vancouver, Canada; e Canadian Advances in Neuro-Orthopedics for Spasticity Consortium, Kingston, Canada

**Keywords:** Cryoneurolysis, Multiple sclerosis, Pain, Rehabilitation

## Abstract

Toe clawing in patients with upper motor neuron disorders is often attributed to the flexor digitorum longus (FDL) and is a common presentation among patients with multiple sclerosis (MS). This movement may be painful because of the altered pressure distribution and may increase the risk of falls, heighten energy expenditure during gait, and lower gait speed. Cryoneurolysis is a minimally invasive treatment that may be beneficial for pain and focal muscle hypertonicity. An ambulatory patient with MS was treated bilaterally with cryoneurolysis to the superficial fibular nerves for pain on the dorsum of the foot, and to the intramuscular tibial nerve motor branch to FDL for toe clawing. The patient felt that toe clawing was immediately reduced during gait and noted the ability to voluntarily spread their toes. The patient stated that the neuropathic pain on the dorsum of the foot was fully eliminated immediately post procedure. The patient reported improved confidence in their gait, maintained independence, and reduced toe clawing during a structured interview 12 weeks after treatment. The effects lasted for 5.5 months before symptoms returned. Retreatment at 6 months reproduced the benefits**.** The patient reported a positive experience with cryoneurolysis for toe clawing and dorsal foot pain.

Patients with multiple sclerosis (MS) are prone to both altered movement patterns and pain.^1^ Characterized by involuntary flexion of the metatarsophalangeal joint, proximal interphalangeal joint, and distal interphalangeal joint, toe clawing is a common lower limb discomfort during gait in patients with MS. Toe clawing is most evident during the stance phase of gait and can increase postural sway and decrease balance, likely increasing the risk of falls. Toe clawing without fixed deformity or spasticity at rest is considered a dystonic pattern.^2^ This unwanted movement causes pain due to the altered distribution of weight through the toes, and the formation of blisters and calluses. Interventions that target the flexor digitorum longus (FDL) may improve ambulation.^3^ A surgical neurolysis of the lateral plantar nerve to address fixed toe clawing has previously been described.^4^

Cryoneurolysis is a minimally invasive procedure that involves the generation of an ice ball at temperatures ranging from -60 to -88 °C depending on the freezing agent. Under ultrasound guidance, a specialized probe used for cryoneurolysis (cryoprobe) is used to create an ice ball from interstitial fluid near a peripheral nerve causing secondary axonotmesis.^5^ The epineurium and perineurium remain intact, providing a pathway for the damaged axon to regenerate.^5^ Recent case studies have shown that increases in range of motion can persist for an extended period, lasting from months to years in numerous etiologies.^6^, ^7^, ^8^ Cryoneurolysis of afferent nerves is an established technique for pain management that has been in use for decades.^9^^,^^10^

## Case report

A 50-year-old woman with MS of more than 20 years had long standing pain on the dorsal aspect of both feet and dystonic toe clawing with associated cramping on standing and walking. There was no appreciable spasticity in the toes with a passive range of motion assessment in the resting position. She has a 10-year history of bilateral botulinum toxin (BoNT) injections to the FDL (50 units of incobotulinum toxin per side). For her neuropathic pain, she used 150 mg of pregabalin 3 times a day, nortriptyline 10 mg every night, and 60 mg of duloxetine daily. The medications assisted with pain in other areas of her body as well. She reported partial relief from toe clawing for up to 10 weeks after BoNT injections, but no change in foot pain. She had reduced vision and used a visual impairment cane at times.

## Methods

This study follows the Case Reports (CARE) guidelines and reports the required information accordingly. Institutional research ethics board approval was not required. Informed consent was obtained for the procedures and for the preparation of this manuscript.

### Clinical evaluation and diagnostic nerve block

**Fig 1:**
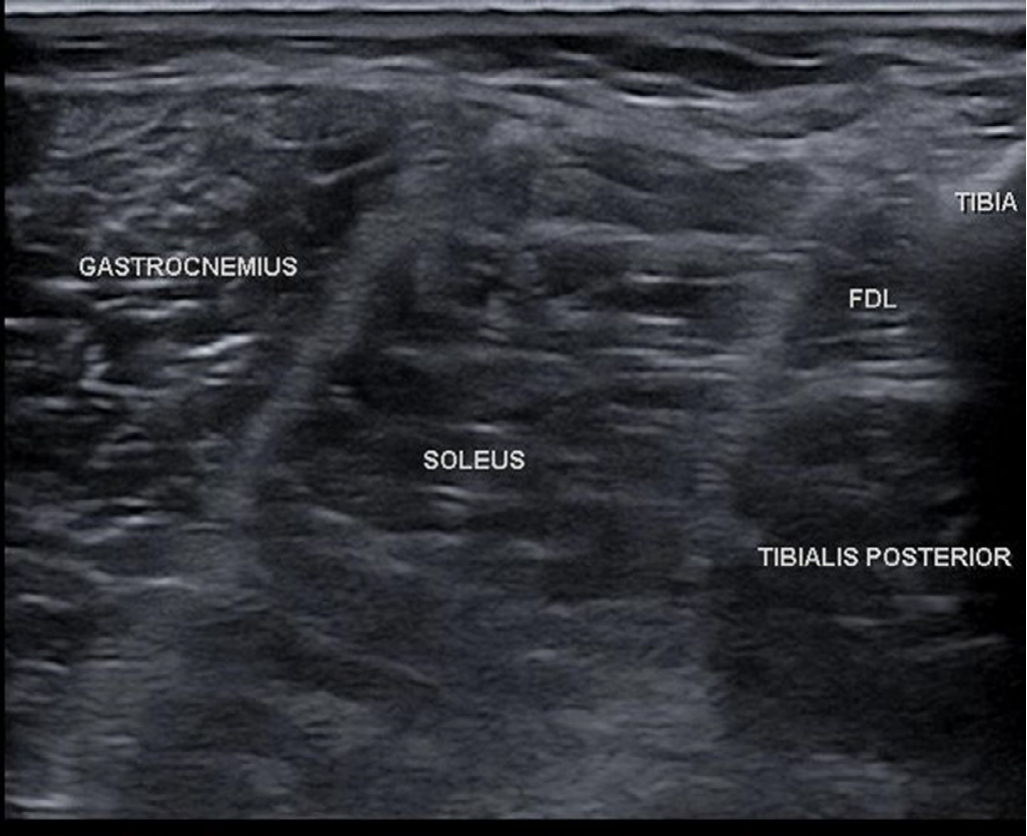
Ultrasound anatomy of the right flexor digitorum longus. The FDL is hypoechoic region deep to the soleus and superficial to the tibialis posterior.

**Fig 2:**
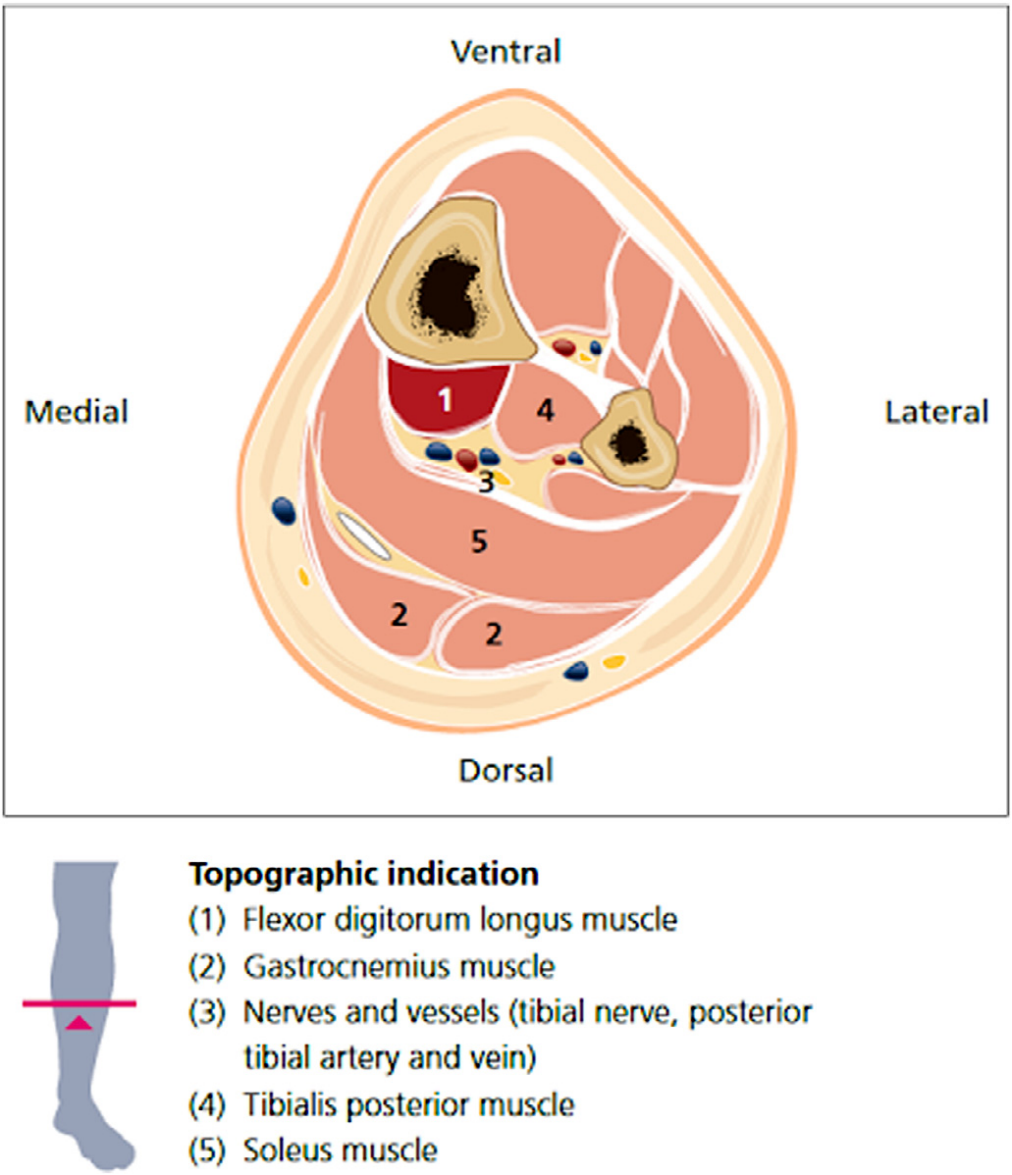
Graphical depiction of the anatomy of the lower leg. Figure from “Atlas of Ultrasound Guided Nerve Targeted Procedures for Spasticity.” Winston & Vincent. Quintessence, Berlin, 2023; p 169.^11^ Reproduced with permission.

On clinical examination, the FDL was determined to be implicated in the toe clawing and was therefore identified as a potential target for cryoneurolysis. The flexor hallucis longus was determined not to be a significant contributor. The tibial nerve intramuscular branch to FDL was visualized on ultrasound; electrical stimulation was used to confirm the target (refer to Fig 1, Fig 2). A diagnostic nerve block (DNB) was performed to the FDL on the right side with an injection of 1 cc of 2% lidocaine.^12^

**Fig 3:**
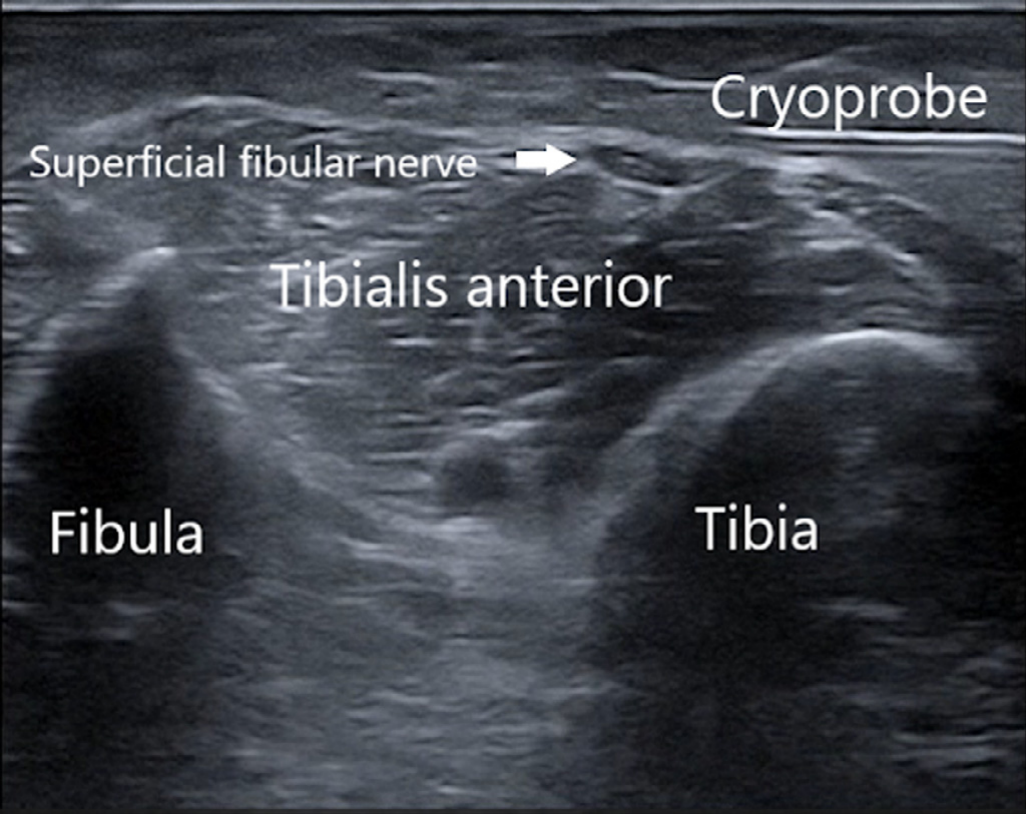
Periprocedural ultrasound image of superficial fibular nerve cryoneurolysis. The cryoprobe is in the top right section of the image, approaching the superficial fibular nerve which lies superficial to the tibialis anterior.

Sensory innervation to the dorsum of the foot is provided by the superficial fibular nerve. This pain generator was chosen as the target to address the patient's primary pain. The superficial fibular nerve was visualized on ultrasound and a DNB was performed to hydro dissect the nerve 15 cm distal to the fibular head (refer to fig 3).

There was no undesirable loss of sensation or function because of the DNBs; no adverse events were reported during or immediately after the treatment.

### Cryoneurolysis

After the unilateral DNBs were performed, the patient noted immediate improvement in pain and relaxation of the toes. The patient was warned of potential side effects and adverse events and consented to bilateral cryoneurolysis of the same targets using the Iovera Handheld System.^a^ This is a free-standing unit that uses liquid nitrous oxide capsules. After the effects of the lidocaine waned, the skin around the injection site was prepared with chlorhexidine swabs to lower the risk of infection. The entry point was anesthetized with a local infiltration of 1% lidocaine. A 16-gauge angiocatheter was inserted to guide the cryoprobe, to increase ultrasound echogenicity, and to protect the skin from potential cold-related adverse events. The cryoprobe was inserted through the catheter and the target nerve was located using known anatomy, as established during the DNB. For the intramuscular motor branch to the FDL, the target was confirmed prior to each cycle using electrical stimulation of less than 1 mA at 1 Hz. Two lesions were created along the intramuscular FDL branches (figs 1 and 2), and 2 lesions above and below the superficial fibular nerve (fig 3). Each of the 4 lesions on each side involved a 106 second freezing and thawing cycle. Refer to the supplemental video S1 (available online only at http://www.archives-pmr.org/)for a brief demonstration of the procedure.

## Results

After cryoneurolysis, the patient described having immediate pain relief to the top of her foot, was able to wiggle her toes, and reported an improvement in her gait due to reduced toe clawing. She reported a tolerable burning pain during the procedure, which was forewarned to the patient. There was mild residual pain and bruising around the injection site immediately after the procedure, which was otherwise well-tolerated. No adverse events were reported. After 10 weeks, the patient reported a sustained reduction in toe-curling, which she felt improved her gait. The pain to the dorsum of the foot had not returned since treatment. An interview with the patient was conducted 12 weeks after treatment and is reported in table 1. She returned to clinic at 6 months, noting that the pain and toe clawing had returned 2 weeks prior and requested retreatment. There was similar bilateral improvement in both parameters, at the 1-month follow-up after the retreatment.

**Table 1:** Summary of the interview with the patient 12 weeks after treatment

How does your experience with botulinum toxin injections compare with your experience with cryoneurolysis?	“Cryoneurolysis is more painful and scary [than botulinum toxin] because of the big needle but the amazing results outweigh [that] completely”
Did you find that the cryoneurolysis procedure was painful?	“Any pain I experienced was completely overshadowed by how amazing I felt right after.”
Have you noticed any side effects from the procedure?	“It was quite tender, but it resolved, I wore compression socks for about a week afterward and that was helpful. Advil (ibuprofen) and Voltaren gel (diclofenac) were also helpful for the pain.”
Did you notice any changes immediately after the cryoneurolysis?	“Almost immediately, when I got off the bed, it was clear that the pain had improved right away, and I had increased mobility. We all had a hug and a cry. It's just remarkable this treatment really. I lifted my toes up off the floor for the first time in 20 years”
Have any family members or friends noticed a difference since the procedure?	“As the weeks passed, I had even more increased mobility and decreased pain. At Christmas, I got to show my friends and family my new party trick where I get up off the floor by extending my toes and pushing into the ground. I hadn't been able to do this for 20 years”
How are you managing your activities of daily living?	“I used to trip because of her toe curling but since the procedure, I am tripping less and managing more easily.”
Is there anything that you could not do before the procedure that you can do now?	“Yes. Since December I've been able to go hiking in the woods. The toe clawing and poor balance had always stopped me from hiking on uneven surfaces.”
Do you have any suggestions on how to improve the experience?	“No suggestions. I just want everyone to know that even though it appears scary, it is so worth it.”
Are you happy with your results from the procedure?	“Extremely. It's really a miracle treatment. It was a Christmas gift. To have this treatment give me this much improvement is amazing.”

## Discussion

This paper is the first report, as far as we know, to describe a patient's report of improved gait and reduced neuropathic pain on the dorsal aspect of the feet after bilateral cryoneurolysis of the FDL motor branch and superficial fibular nerve. The challenges of addressing both toe clawing and neuropathic pain in MS have been described: Deltombe et al^13^ opined that the nerve branches that travel to the FDL are mixed with the sensory fibers which also innervate the sole of the foot, and they recommended avoiding a surgical neurotomy for this muscle and treating the claw toe by means of tendon surgery. We avoided altering the proprioceptive fibers of the tibial nerve to the sole of the foot by doing a selective, intramuscular cryoneurolysis. Urits et al^1^ noted that neuropathic pain in MS patients is frequently unremitting and responds poorly to conventional treatments, often requiring novel interventions.

The FDL is a deep muscle that inserts proximally on the posterior surface of the tibia, inferior to the soleal line. Distally, the FDL tendon enters the sole of the foot near the sustentaculum tali, crosses the tendon of the flexor hallucis longus and then attaches onto the bases of the second through fifth distal phalanges. The FDL is innervated by the L5-S1-S2 contributions of the tibial nerve and contributes to plantar flexion of the foot, and flexion at the metatarsophalangeal joint, proximal interphalangeal joint, and distal interphalangeal joint of toes 2 through 5.^14^ The tibial nerve provides motor innervation to the muscles of the posterior compartment of the leg, including the gastrocnemius, soleus, tibialis posterior, flexor hallucis longus, FDL, and the intrinsic muscles of the foot.^15^ Clinical examination did not find that intrinsic muscles were implicated in the claw toe.

The superficial fibular nerve originates from the dorsal branches of the ventral rami of nerve roots L4, L5, S1, and S2. The nerve descends into the lateral compartment of the leg, medial to the biceps femoris, and between the bicipital tendon and the lateral head of the gastrocnemius. It then travels through the deep fascia overlying the tibialis anterior and curves lateral to the fibular neck where it divides into the superficial and deep fibular nerves. The nerve innervates both the fibularis longus and brevis. The sensory component of the superficial fibular nerve splits into medial and lateral branches anterior to the ankle and innervates the dorsum of the foot, except for the first web space.^16^

Previous studies show that cryoneurolysis of sensory neurons may provide long-lasting pain relief in multiple different conditions.^9^^,^^10^^,^^17^ Our patient noted five and half months of relief of pain and toe clawing. The present case study is the first to demonstrate cryoneurolysis of the superficial fibular nerve as a potential technique for the management of neuropathic pain on the dorsum of the foot in an ambulatory patient with MS.

Toe clawing and neuropathic pain are common afflictions among patients with MS who are often not fully resolved with conventional therapies.^1^^,^^18^ There are currently no validated objective assessment techniques for dystonic toe clawing. Baccouche et al^18^^,^^19^ noted that only 79% of patient goals for toe clawing or clinging are likely to be achieved compared with 88% in other presentations in patients with MS when treated with BoNT.

A concern of neurolysis in an ambulatory patient is that the treatment will lead to undesirable weakness or sensory loss that can worsen gait. This type of adverse event is likely to stem from spread of the neurolytic agent away from the targeted structures, poor localization of target nerves, or poor specificity of treatment. Cryoneurolysis involves the formation of a cold zone and does not inject any liquid agent; there is no risk of liquid spreading to untargeted nerves. Ultrasound guidance and electrical stimulation (for motor targets) allows for extremely precise localization and treatment that further reduces the risk of undesirable weakness or sensory loss from poor localization. Finally, the use of a DNB prior to cryoneurolysis likely reduces the risk of adverse events from poor specificity by informing treatment decisions before any long-lasting intervention is applied. This process complements the clinical evaluation to ensure that only nerves innervating muscles that are directly implicated in the pathology are treated.

Cryoneurolysis has been well tolerated by most patients with adverse events being described as mild in severity.^19^ A recent secondary cohort analysis of patients treated with cryoneurolysis of mixed and primarily motor nerves showed side effects occurring after only 1% of treatments for primarily motor nerves and none for intramuscular nerve branches.^20^ The most frequently reported adverse events are dysesthesia and paresthesia. In this case, numbness was a positive outcome as it provided the patient pain relief.

## Limitations

The described case reports on the experience of a single patient and is not generalizable to the entire population of patients with MS with toe clawing and neuropathic pain; MS is a progressive and diverse illness that is unique to each patient, leading to varying responses to treatment. Further research should validate objective measures of treatment efficacy for dystonic toe clawing. Future studies should include well-designed randomized controlled trials to understand the efficacy of this treatment compared with other options.

## Conclusions

Bilateral cryoneurolysis of the tibial nerve intramuscular branch to FDL, and of the superficial fibular nerve were performed for toe clawing and dorsal foot pain in a patient with MS. The patient reported a positive experience with cryoneurolysis when interviewed 12 weeks after treatment, which lasted to five and half months before retreatment was required. Further studies are required to understand the efficacy of this treatment.

## Suppliers

a. Iovera 190 Smart Tip; Pacira Bioscicences

## Appendix Supplementary materials

Image, video 1

## Footnotes

Supplementary material associated with this article can be found, in the online version, at doi:10.1016/j.arrct.2023.100286.

## References

[1] Urits I, Adamian L, Fiocchi J (2019). Advances in the understanding and management of chronic pain in multiple sclerosis: a comprehensive review. Curr Pain Headache Rep.

[2] Verdié C, Daviet JC, Borie MJ (2004). [Epidemiology of pes varus and/or equinus one year after a first cerebral hemisphere stroke: apropos of a cohort of 86 patients]. Ann Readapt Med Phys.

[3] Lim ECH, Ong BKC, Seet RCS. (2006). Botulinum toxin-A injections for spastic toe clawing. Parkinsonism Relat Disord.

[4] Dellon AL, Steck JK. (2008). Reversal of toe clawing in the patient with neuropathy by neurolysis of the distal tibial nerve. Microsurgery.

[5] Gage AA, Baust JM, Baust JG. (2009). Experimental cryosurgery investigations in vivo. Cryobiology.

[6] MacRae F, Brar A, Boissonnault E, Winston P. (2023). Cryoneurolysis of anterior and posterior divisions of the obturator nerve. Am J Phys Med Rehabil.

[7] Rubenstein J, Harvey AW, Vincent D, Winston P. (2021). Cryoneurotomy to reduce spasticity and improve range of motion in spastic flexed elbow: a visual vignette. Am J Phys Med Rehabil.

[8] Winston P, Mills PB, Reebye R, Vincent D. (2019). Cryoneurotomy as a percutaneous mini-invasive therapy for the treatment of the spastic limb: case presentation, review of the literature, and proposed approach for use. Arch Rehabil Res Clin Transl.

[9] Trescot A. (2003). Cryoanalgesia in interventional pain management. Pain Physician.

[10] Ilfeld BM, Gabriel RA, Trescot AM. (2017). Ultrasound-guided percutaneous cryoneurolysis providing postoperative analgesia lasting many weeks following a single administration: a replacement for continuous peripheral nerve blocks?: a case report. Korean J Anesthesiol.

[11] Winston P, Vincent D. (2023).

[12] Winston P, Reebye R, Picelli A, David R, Boissonnault E. (2023 Feb 4). Recommendations for ultrasound guidance for diagnostic nerve blocks for spasticity. What are the benefits?. Arch Phys Med Rehabil.

[13] Deltombe T, Wautier D, De Cloedt P, Fostier M, Gustin T. (2017). Assessment and treatment of spastic equinovarus foot after stroke: guidance from the Mont-Godinne interdisciplinary group. J Rehabil Med.

[14] Lavallee JR, Pourcho AM, Henning PT, Lambert HW. (2017). Sonographic identification of the flexor digitorum accessorius longus tendon. PM&R.

[15] Desai SS, Cohen-Levy WB. (2022). https://www.ncbi.nlm.nih.gov/books/NBK537028/.

[16] Garrett A, Geiger Z. (2022). https://www.ncbi.nlm.nih.gov/books/NBK534793/.

[17] Gabriel RA, Finneran JJ, Asokan D, Trescot AM, Sandhu NS, Ilfeld BM. (2017). Ultrasound-guided percutaneous cryoneurolysis for acute pain management: a case report. A Case Rep.

[18] Baccouche I, Bensmail D, Leblong E (2022). Goal-setting in multiple sclerosis-related spasticity treated with botulinum toxin: the GASEPTOX study. Toxins.

[19] Perry TA, Segal NA. (2022). An open-label, single-arm trial of cryoneurolysis for improvements in pain, activities of daily living and quality of life in patients with symptomatic ankle osteoarthritis. Osteoarthr Cartil Open.

[20] Winston P, MacRae F, Rajapakshe S (2023 Apr 25). Analysis of side effects of cryoneurolysis for the treatment of spasticity. Am J Phys Med Rehabil.

